# Telehealth Interventions for Improving Self-Management in Patients With Hemophilia: Scoping Review of Clinical Studies

**DOI:** 10.2196/12340

**Published:** 2019-07-10

**Authors:** Wenji Qian, Teddy Tai-Ning Lam, Henry Hon Wai Lam, Chi-Kong Li, Yin Ting Cheung

**Affiliations:** 1 School of Public Health Fudan University Shanghai China; 2 Key Laboratory of Health Technology Assessment National Health Commission of the People’s Republic of China Shanghai China; 3 School of Pharmacy Faculty of Medicine The Chinese University of Hong Kong Hong Kong China (Hong Kong); 4 The Hong Kong Haemophilia Society Hong Kong China (Hong Kong); 5 Department of Paediatrics, Faculty of Medicine The Chinese University of Hong Kong Hong Kong China (Hong Kong); 6 Paediatric Haematology & Oncology Hong Kong Children's Hospital Hong Kong China (Hong Kong)

**Keywords:** hemophilia, clotting factors, adherence, self-management, telehealth

## Abstract

**Background:**

The introduction of home therapy for hemophilia has empowered patients and their families to manage the disease more independently. However, self-management of hemophilia is demanding and complex. The uses of innovative interventions delivered by telehealth routes such as social media and Web-based and mobile apps, may help monitor bleeding events and promote the appropriate use of clotting factors among patients with hemophilia.

**Objective:**

This scoping review aims to summarize the literature evaluating the effectiveness of telehealth interventions for improving health outcomes in patients with hemophilia and provides direction for future research.

**Methods:**

A search was conducted in Ovid MEDLINE, EMBASE, and PubMed databases for studies that (1) focused on patients with hemophilia A or B; (2) tested the use of remote telehealth interventions via the internet, wireless, satellite, telephone, and mobile phone media on patients and caregivers; and (3) reported on at least one of the following patient-/caregiver-focused outcomes related to empowering patients/caregivers to be active decision makers in the emotional, social, and medical management of the illness: quality of life, monitoring of bleeding episodes, joint damage or other measures of functional status, medication adherence, and patients’ knowledge. Implementation outcomes (user metrics, cost saving, and accuracy of electronic records) were also evaluated. Reviews, commentaries, and case reports comprising ≤10 cases were excluded.

**Results:**

Sixteen articles fulfilled the inclusion criteria. The majority of the interventions (10/16, 62%) evaluated both implementation outcomes and patient-/caregiver-focused outcomes. User performance and accuracy and comprehensiveness of electronic records were also measured in most studies (4/16, 87%). The components of the interventions were rather homogenous and typically involved electronic logging and reminders for prophylactic infusions, reporting of spontaneous and traumatic bleeding events, monitoring of infusion product usage and home inventory, and real-time communication with health care professionals and hemophilia clinics. Telemedicine-supported education and information interventions seemed to be particularly effective among adolescent and young adult patients. Although the patients reported improvements in their health-related quality of life and perception of illness, telemonitoring devices did not appear to have a significant effect on quantifiable health outcomes such as joint health. Longitudinal studies seemed to suggest that the response and adherence rates to recording decreased over time.

**Conclusions:**

Preliminary evidence from this review suggests that telehealth-delivered interventions could feasibly improve patients’ adherence to medication use and promote independence in disease management. Given the complexity and resources involved in developing a mature and established system, support from a dedicated network of hemophilia specialists and data managers will be required to maintain the technology, improve adherence to prophylactic treatment and recording, and validate the electronic data locally.

## Introduction

Hemophilia refers to a rare group of X-linked recessive hemorrhagic diseases that often require complex disease care and treatment. Appropriate management is needed to reduce the bleeding episodes and long-term complications of bleeding, such as chronic arthropathy and intracerebral hemorrhage, as well as the frequency of hospitalization and absenteeism from school or work [1]. Fortunately, the introduction of home therapy for hemophilia has empowered patients and their families to manage the disease more independently. However, the self-management of hemophilia is demanding, including the recognition of bleeding, adherence to self-administration of prophylactic infusions, recording of bleeding events, and management of a home inventory of medications.

Strategies are needed to improve the health outcomes and self-efficacy of patients with hemophilia. To address these needs, platforms involving different types of technology have been implemented to promote health education and good protective health behavior. The Health Resources and Services Administration defines the term “telehealth” as the use of electronic information and telecommunications technologies to support and promote long-distance clinical health care, health-related education for patients and professionals, public health, and health administration [2]. For the purpose of this review, we have broadly defined “telemedicine” or “telehealth intervention” as interventions that use telecommunications technology to facilitate the remote delivery of health care services and clinical information [2,3]. These interventions can be synchronous or asynchronous and include any information and technology-based strategies for connecting health care professionals and patients through video conferencing, e-mail, remote electronic monitoring equipment, social network apps, and internet portals [3]. By nature, telehealth interventions include interactive telemedicine services that facilitate concurrent interactions among patients, caregivers, and clinicians, and the remote monitoring of patients’ health statuses using telehealth equipment and “store-and-forward telemedicine,” which involves the transmission of disease-related data such as medical images, bleeding, episodes, and biological measures [4]. As adherence to the recording of bleeding episodes and infusion plays an important role in treatment outcomes, applications of telehealth intervention in hemophilia include the use of an electronic device to collect information on bleeding episodes and real-time transmission to the hemophilia clinic or the use of videoconferencing to educate patients on infusion techniques in a remote setting [4].

The literature includes a preponderance of reviews that discuss the benefits and challenges of interventions in the management of chronic diseases delivered via telehealth [4-6]. Emerging evidence supports the use of technology to facilitate the self-management of complex and demanding treatments for chronic diseases. The uses of innovative interventions delivered by telehealth routes, such as social media, mobile apps, and teleconferences, may help monitor bleeding events and promote adherence in prophylactic infusion of clotting factors among patients with hemophilia through built-in alarms and reminders [4]. This delivery route may also be harnessed to increase patients’ motivation for participating in self-care activities and protective health behaviors in terms of the recommended diet and exercise. Although previous systematic reviews have focused on the applicability of interventions delivered by telehealth for patients with chronic conditions, to our knowledge, none have involved the population of patients with hemophilia. This review aims to summarize the literature evaluating the effectiveness of telehealth interventions for improving health outcomes in patients with hemophilia and to provide directions for future research.

## Methods

### Overall Approach

In the initial phase of this study, the original protocol was developed as a systematic review to quantitatively address the feasibility, appropriateness, and effectiveness of telehealth intervention in patients with hemophilia. However, a preliminary search revealed high heterogeneity in the assessment of health outcomes across studies with limited sample sizes and generalizability. Consensus among the investigators resulted in a protocol modification to perform a scoping review instead. We rationalized that this is a reasonable decision because scoping reviews follow a narrative synthesis approach, which is more appropriate for summarizing review studies that vary in methodological approaches.

We followed the steps recommended by Arskey and O’Malley [7] to perform the review: (1) identify the research questions; (2) identify relevant studies; (3) select studies; (4) chart the data; and (5) collate, summarize, and report the results of the included studies. To ensure transparency and complete reporting, we followed the PRISMA extension (PRISMA-ScR) for scoping reviews [8].

### Search Procedure

**Figure 1:**
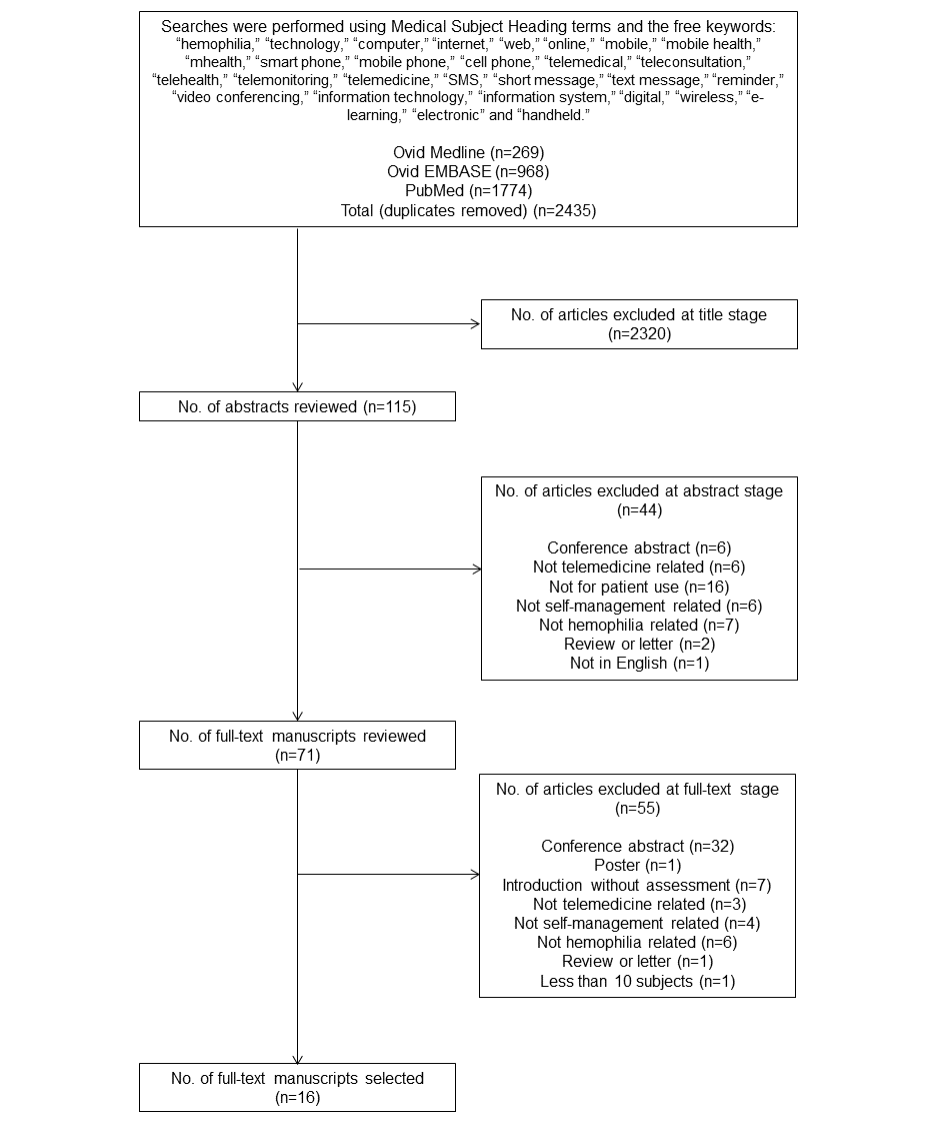
Flowchart of the literature search.

The Ovid MEDLINE, EMBASE and PubMed databases were searched in July 2018 using the following combination of terms: “hemophilia,” “technology,” “computer,” “internet,” “web,” “online,” “mobile,” “mobile health,” “mhealth,” “smart phone,” “mobile phone,” “cell phone,” “telemedical,” “teleconsultation,” “telehealth,” “telemonitoring,” “telemedicine,” “SMS,” “short message,” “text message,” “reminder,” “video conferencing,” “information technology,” “information system,” “digital,” “wireless,” “e-learning,” “electronic” and “handheld.” Only English-language articles published before July 31, 2018, were reviewed. The search strategies comprised Medical Subject Headings, keywords, and text words related to bleeding disorders and telehealth. The complete search strategies are provided in  Figure 1. We also manually searched the reference lists of the retrieved manuscripts.

The inclusion and exclusion criteria were as follows: studies focusing on patients with hemophilia A or B; studies testing the use of remote telehealth interventions via the internet, wireless, satellite, telephone, and mobile phone media on the patient or caregiver of the patient; and studies reporting on at least one of the following primary outcomes related to empowering patients or caregivers to be active decision makers in the emotional, social, or medical management of their illness or the child’s illness. These outcomes were identified from the literature as critical for improving patient efficacy in the management of hemophilia [9] as well as by clinical consensus from the team of investigators comprising methodologists (WQ and YC), a hematologist (CL), a pharmacist (TL), and a representative of a support group for patients with hemophilia (HL). Examples of such outcomes included quality of life; monitoring and adherence to the recording of bleeding episodes; adherence to prophylactic infusion; joint damage or other measures of functional status; medication adherence; patient knowledge; and patients’ perceived value, acceptance, and satisfaction with the intervention. Secondary outcomes related to the development and implementation of the intervention were also evaluated. These outcomes included, but were not limited to, user metrics, the comparison of reliability and accuracy between electronic and paper records, and impact of intervention on cost and resource saving.

The exclusion criteria were as follows: (1) reviews, commentaries, conference proceedings, dissertation reports, or case reports comprising 10 or fewer cases and (2) studies that did not describe the basic quantitative or qualitative research methodology such as data collection methods, clinical assessment methods and definitions, and analytic or reporting strategies.

### Data Extraction and Analysis

The search results were reviewed on three sequential levels: (1) In the initial “title stage,” the article titles were screened to exclude studies that clearly did not fulfil the inclusion criteria outlined in this review. (2) In the “abstract stage,” the abstracts of articles that passed the “title stage” were reviewed. (3) In the final “full-text stage,” the remaining articles were examined to ensure that they fulfilled the inclusion/exclusion criteria. The screening and data extraction phases were conducted by the investigators (WQ and YC) independently. The list of included studies and summary of data prepared by the two investigators were then compared, and disagreements were resolved through discussion and involvement of a third investigator (TL). The study characteristics were systematically abstracted using a structured data collection form that included the following parameters: the country in which the study was conducted, year of publication, study design, sample size, patient characteristics, description of intervention, assessment outcomes, and tools and conclusion.

### Quality Assessment

Although quality assessment of the included studies is generally not required for a scoping review, assessment of the methodological limitations was evaluated to establish the quality of existing evidence and address variation in the study approaches. Two reviewers assessed the methodological quality of the observational studies and controlled trials (WQ and YC) using the Effective Public Health Practice Project Quality Assessment Tool for Quantitative Studies (EPHPP) [10]. Any discrepancies in the ratings were resolved by discussion. Interrater reliability was calculated using the Cohen kappa statistic to ascertain the agreement between the scores of the two reviewers in terms of each criterion of the EPHPP [11]. The EPHPP determines the quality of each study based on six criteria: selection bias, study design, confounders, blinding, data collection methods, and withdrawals/dropouts. Each individual component was ranked as strong, moderate, or weak according to the EPHPP. Finally, the sum of all six criteria was calculated to construct a global rating that represents the overall robustness and quality of the study methodology. Two additional EPHPP components—integrity and consistency of the intervention and the appropriateness of analytical methods—are independent scales that are not included in the overall grading [10].

## Results

### General Characteristics

The results of the literature search are depicted in  Figure 1. The search resulted in 2435 titles. After comparing the titles and abstracts against the eligibility criteria, 2364 articles were excluded, leaving 71 articles for full-text screening. After an independent review of the full texts, 16 articles fulfilled the inclusion criteria [12-27]. The general characteristics of these studies are presented in Table 1.

Of the 16 articles, three (19%) described randomized controlled trials (RCTs) [14,25,26], two (12%) described qualitative studies [15,27], and the remaining (n=11, 69%) described observational studies [12,13,16-24]. Eight studies (50%) were conducted in North America [14,15,19,20,22,23,26,27]; five (31%), in Europe [16-18,24,25]; two (12%), in the United Kingdom [12,13]; and one (6%), in Australia [21].

The sample sizes of the studies were generally small, ranging from 10 to 50 patients. Broderick et al [21] investigated the response rate and expenses incurred from implementing a short message service (SMS) intervention in a cohort of 104 patients with hemophilia in Australia [21]. One large-scale study recruited 2683 patients from different regions of the United Kingdom to evaluate usage metrics and compliance with a nationwide electronic recording platform [13]. The majority of studies involved patients with severe hemophilia who required prophylactic infusion therapy. The age distributions varied widely across the studies, which include both adult patients and caregivers of pediatric or adolescent patients. Two studies specifically targeted the delivery of educational resources regarding self-management of adolescents between the ages of 12 and 18 years [26,27].

**Table 1:** Characteristics of patients in the included studies.

Study and year	Country	Design^a^	Sample size (rate)	Disease type (%)	Disease severity	Treatment^b^ (%)	Age (years), proportion or as indicated	Education level
Collins et al, 2003 [12]	United Kingdom	OS^c^ longitudinal	Total: 10	Hem^d^	NR^e^	NR	12-17 years: 70%, 30-45 years: 20%, >50 years: 10%	NR
Walker et al, 2004^f^ [14]	Canada	RCT^g^	Total: 41, test: 22, control: 19, rate: 60%	HemA^h^: 93%, HemB^i^: 7%	Severe: 100%	Prophylaxis: 59%	Median: 25 years (IQR^j^: 15-42 years); <18 years: 63%, ≥18: 37%	NR
Arnold et al, 2005 [15]	Canada	OS/ qualitative	Total: 20	HemA, HemB	Severe: 100%	NR	Range: 6-43	NR
Pattacini et al, 2009 [16]	Italy	OS	Total: 50	NR	NR	NR	NR	NR
Petrini et al, 2009 [17]	Denmark, Finland, Norway, Sweden	OS	Total: 57	HemA: 100%	Moderate: 5%, severe: 95%	All on ReFacto, on demand: 19%, prophylaxis: 81%	Median (range): 17.5 (0.4-57.2)	NR
Mondorf et al, 2009 [18]	Germany	OS	Total: 10	HemA	Severe: 100%	On demand: 10%, prophylaxis: 90%	Median (range): 31 (11-48)	NR
Vallee-Smejda et al, 2009 [19]	Canada	OS	Total: 18, rate: 47%	Hem	Mild, moderate, severe	NR	Range: 2-67; 16-20: 6%, 21-40: 44%, 41-60: 50%	High school: 33%, college: 22%, undergraduate: 28%, doctorate: 6%
Leone et al, 2011 [20]	United States	OS	Total: 52, rate: 100%	HemA, HemB	NR	Prophylaxis: 100%	All >5 years	NR
Broderick et al, 2012 [21]	Australia	OS	Total: 104	HemA, HemB	Moderate, severe	NR	Mean (SD): 9.5 (4.0); range: 4-18	NR
Mulders et al, 2012 [25]	The Netherlands	RCT	Total: 30, test: 16, control: 14, rate: 76%	HemA: 77%, HemB: 23%	Mild: 3%, moderate: 36%, severe: 60%	Prophylaxis: 40%, on demand: 26%, both: 34%	Median (range): 33.5 (17-67)	Elementary: 6%, secondary: 43%, vocational advanced: 36%, academic: 4 (13%)
Young et al, 2012 [22]	United States	OS	Total: 52	HemA, HemB	≥4 bleeding events in the prior 3 months	On demand: 71%, ITT^k^: 10%, prophylaxis: 23%	<18: 48%, ≥18: 52%	NR
Breakey et al, 2013 [27]	Canada	OS/qualitative	Total: 18, rate: 80%	HemA: 67%, HemB: 22%, Unsure: 11%	Mild: 17%, moderate:17%, severe: 66%	Prophylaxis: 78%	Median (range): 15.5 (13-18)	English speaking in grade 9-12: 67%, French speaking in grade 3-5: 33%
Sholapur et al, 2013 [23]	Canada	OS	Total: 51, test: 23, others: 28, rate: 62%	NR	NR	NR	<16: 39%, ≥16: 61%	NR
Breakey et al, 2014 [26]	Canada	RCT	Total: 29, test: 16, control: 13	HemA: 62%, HemB: 31%, Unsure: 7%	Mild: 21%, moderate: 24%, severe: 55%	Prophylaxis: 76%, inhibitor: 4%	Mean (SD): 15.9 (1.34); range: 13-18	Grade 10 (median)
Hay et al, 2017 [13]	United Kingdom	OS	Total: 2683	HemA: 78%, HemB: 15%, Others: 7%	Mild: 9%, moderate 10%, severe: 81%	NR	NR	NR
Cuesta-Barriuso et al, 2018 [24]	Spain	OS longitudinal	Total: 43	HemA: 86%, HemB: 14%	Severe: 91%	Prophylaxis: 100%	Mean (SD): 25.8 (10.3)	Primary: 7%, secondary: 44%, university: 49%

^a^Design: Due to the heterogeneity of methodologies across studies, study designs are classified simply as either randomized controlled trial (RCT) or observational studies for non-RCT studies. Studies that utilized qualitative methods (eg, structured interviews) are specified.

^b^Treatment percentages may not add up to 100% because respondents might have indicated the use of multiple agents.

^c^OS: observational study.

^d^Hem: hemophilia.

^e^NR: not reported.

^f^Patient sample in Arnold, 2005 [15] is a subset of patients from Walker, 2004 [14].

^g^RCT: randomized controlled trial.

^h^HemA: hemophilia A.

^i^HemB: hemophilia B.

^j^IQR: interquartile range.

^k^ITT: immune tolerance treatment.

### Quality of Studies

The assessment of study quality received an interrater agreement *k* of 0.78. Most studies received a “weak” (7/16, 44%) or “moderate” (6/16, 38%) rating, although three studies were considered to be of “strong” methodological quality (Table 2). When considering the individual components of quality, most studies were considered “weak” if they were cross-sectional and single arm in nature and “moderate” or “strong” if they had a controlled trial or cohort design or a pre- and postintervention assessment. Adjustment for confounders is generally not applicable to studies involving only a single arm or those with poorly characterized clinical descriptions of the cohort. A minority of studies (n=2) reported assessments of outcomes blinded to the appropriate members of the research team. Half of the studies applied objective measures (eg, usage metrics, number of diary entries, and cost data) or cited data on the psychometric properties or development methodology behind their instruments of outcome assessments, while others were rated as “weak” if a satisfaction survey was the only mode of evaluation.

**Table 2:** Quality assessment of the included studies. Methodological quality scores of included studies are scored using the “Quality Assessment Tool for Quantitative Studies” developed by the Effective Public Health Practice Project [10].

Study and year	Selection bias	Study design	Confounders	Blinding	Data collection methods	Withdrawals and dropouts	Intervention integrity	Analyses	Global
Collins et al, 2003 [12]	Weak	Moderate	N/A^a^	Weak	Weak	Moderate	Moderate	Weak	Weak
Walker et al, 2004 [14]	Moderate	Strong	Strong	Moderate	Strong	Strong	Moderate	Strong	Strong
Arnold et al, 2005 [15]	Weak	Weak	Weak	Weak	Moderate	N/A	Moderate	Moderate	Weak
Pattacini et al, 2009 [16]	Weak	Weak	N/A	Weak	Weak	N/A	Moderate	Moderate	Weak
Petrini et al, 2009^b^ [17]	Moderate	Weak	N/A	Weak	Weak	Moderate	Moderate	Weak	Weak
Mondorf et al, 2009 [18]	Weak	Weak	N/A	Weak	Weak	Moderate	Weak	Weak	Weak
Vallee-Smejda et al, 2009 [19]	Weak	Moderate	N/A	Weak	Weak	Weak	Moderate	Weak	Weak
Leone et al, 2011 [20]	Moderate	Moderate	N/A	Weak	Weak	Strong	Strong	Weak	Moderate
Broderick et al, 2012 [21]	Moderate	Moderate	Moderate	Weak	Strong	Strong	Moderate	Weak	Moderate
Mulders et al, 2012 [25]	Moderate	Strong	Moderate	Moderate	Moderate	Strong	Moderate	Moderate	Moderate
Young et al, 2012 [22]	Weak	Moderate	N/A	Weak	Moderate	Moderate	Moderate	Moderate	Moderate
Breakey et al, 2013 [27]	Moderate	Moderate	N/A	Weak	Moderate	N/A	Moderate	Moderate	Moderate
Sholapur et al, 2013 [23]	Weak	Weak	Weak	Weak	Weak	N/A	Weak	Moderate	Weak
Breakey et al, 2014 [26]	Moderate	Strong	Moderate	Weak	Strong	Strong	Moderate	Strong	Strong
Hay et al, 2017 [13]	Moderate	Weak	Strong	Weak	Strong	N/A	Moderate	Strong	Strong
Cuesta-Barriuso et al, 2018 [24]	Moderate	Moderate	N/A	Weak	Strong	Moderate	Strong	Strong	Moderate

^a^N/A: not applicable.

### Intervention Characteristics and Outcomes

Intervention characteristics and outcomes of the included studies are presented in Table 3. The majority of the interventions (10/16, 62%) evaluated both implementation outcomes and patient-/caregiver-focused outcomes. The most commonly adopted patient-/caregiver-focused outcomes included joint health, adherence to prophylactic treatment, health-related quality of life, and self-management skills. User performance and accuracy and comprehensiveness of electronic records were also measured in most studies (14/16, 87%).

The components of the interventions were rather homogenous and typically involved electronic logging and reminders for prophylactic infusions, electronic reporting of spontaneous and traumatic bleeding events, electronic monitoring of infusion product usage and home inventory, and electronic real-time communication with health care professionals and hemophilia clinics. The use of electronic diaries in the form of handheld computers and Web-based apps was the most common mode of intervention. However, the findings were rather inconsistent and depended on the outcome of interest. Narrative syntheses found that electronic diaries seemed to improve patient adherence and accuracy when recording bleeding episodes and infusion logs [14,15,19,24]. Using a validated scale for measuring patient’s adherence to prophylactic treatment, Cuesta-Barriuso et al [24] reported a statistically significant decrease in adherence problems in patients at 12 months from the initiation of the Medtep Hemophilia online platform (baseline mean score: 44.6 [SD 10.4]; 12-month postintervention mean score: 33.6 [SD 6.5]; *P*<.001). The same study found a statistically significant improvement in most quality of life components on the Short Form-36, such as general health (baseline mean score: 48.4 [SD 9.3]; 12-month score: 57.1 [SD 5.6]; *P*<.001) and body pain (baseline mean score: 49.9 [SD 7.6]; 12-month score 52.8 [SD 6.3]; *P*<.05) [24]. Vallee-Smejda et al [19] demonstrated that the amount of completed additional fields nearly doubled with the use of an electronic diary, indicating improvement in the completeness of data entry. In terms of accuracy, one study reported a reasonably good agreement of 93% between the electronic records and paper diaries [22]. Leone et al [20] also emphasized how patients viewed the importance of the image-taking features of their monitoring devices for the purpose of capturing photographs of their bleeding joints.

However, longitudinal studies seemed to suggest that the rate of adherence to electronic reporting decreased over time [13,17]. For example, the response rate of reporting bleeding events through an SMS intervention decreased from approximately 95% at initiation to 85% after 1 year [21]. Petrini et al [17] reported that a decrease in usage of electronic recording was largely attributed to technical problems and the challenges involved in correcting errors that required contact with the primary clinics. Although the patients reported improvements in their health-related quality of life and perception of illness, telemonitoring devices did not appear to have a significant effect on quantifiable health outcomes such as joint health [24]. The assessment of such long-term indicators may have been limited by the short time horizons of the included studies, which ranged from only 8 weeks to 1 year. Patients who relied solely on on-demand treatments did not report any benefit of an electronic documentation system [18].

Three studies that focused on the provision of disease-related information and practical skills regarding the management of hemophilia yielded promising outcomes [25-27]. Specifically, these studies implemented a robust methodology with appropriate randomization strategy and analytic methods that account for confounding factors. Mulders et al [25] reported that patients who engaged in a 1-month educational electronic learning program demonstrated significantly higher levels of theoretical knowledge on hemophilia, bleeding treatment, and complications of treatment than control subjects (mean score: 75% vs 54%; *P*<.001) as well as better skills in the intravenous injection of clotting factor concentrates (*P*<.001). Telemedicine-supported education and information interventions seemed to be particularly effective among adolescent patients [26,27]. Breakey et al [26] evaluated the feasibility of an internet-based self-management and transitional care program for adolescents with hemophilia. They found that, compared to controls, adolescents in the intervention arm showed a significant improvement in disease-specific knowledge (*P*=.004), self-efficacy (*P*=.007), and transition preparedness (*P*=.046) using a structured questionnaire [26]. In addition, adherence to the completion of the final online outcome measures revealed that 17 of 18 (94%) teenagers successfully completed all the postprogram outcome measures.

**Table 3:** Characteristics of telehealth interventions and main outcomes.

Study	Intervention features	Patient-/caregiver-focused outcomes	Implementation and intervention-focused outcomes	Findings
Collins et al, 2003 [12]	Advoy.com (internet-based electronic patient treatment record and communication system) Documented bleeding event, infusion log, and symptoms Triggers and alerts can be set by clinicians Patient contacted by phone Duration: 8 weeks	Perceived value of intervention	Comparison of electronic and previous paper treatment records	Reported electronic recording to be easier for treatment log: 8 patients (80%) Reported electronic recording to be worse for treatment log: 2 patients (20%) Reported electronic treatment log to be more accurate: 9 patients (90%)
Walker et al, 2004 [14]	Dialog: Electronic diary Handheld computer Documented infusion, bleeding event, symptoms, and productivity Data transmitted to clinic Bar code reader for medications Single reminder phone call at the end of every month Preintervention training for patients Duration: 6 months	—^a^	Usage metrics Accuracy and comprehensiveness of data	86.2% of infusions by patients in the intervention group were adherent with the data submission schedule, as compared to only 48.3% in the control group The time intervals between infusions and the receipt of data were shorter in the intervention group (median 0.25 days) as compared to the control group (25 days). Accuracy of data was similar for both methods. Reminder phone calls by the clinic made less frequently to the intervention group (median: 1 time/month) as compared to the control group (median: 5 times/month)
Arnold et al, 2005 [15]	Dialog: Electronic diary Handheld computer (as in [14])	Patients’ preferred choice of recording method: paper diaries versus handheld computers through semistructured interviews	Usage metrics	All patients preferred using handheld computers to using paper diaries 90% believed that their adherence to record keeping had improved using handheld computers
Pattacini et al, 2009 [16]	“xl_Emofilia” (Web-based app) Record bleeding events and home infusions Collaborating sites have access to patient data Preintervention training for patients	Level of patient satisfaction	Usage metrics Degree of accuracy from validation	825 log-ins made 105 bleeding episodes or traumatic events recorded Degree of accuracy: 80% in the first month and 95% in the subsequent period High degree of acceptance among patients
Petrini et al, 2009^b^ [17]	Electronic diary Handheld computer Documented bleeding event	—	Usage metrics	Adherence was lower than expected, with ≤50% reporting accurately during the entire study period. Some patients reported a large number of infusions from a long time period, on one day. Technical problems were a major contributing factor to poor adherence. Examination of the diary data revealed useful information on the management of bleeding episodes.
Mondorf et al, 2009 [18]	Haemoassist (handheld electronic diary) Documented bleeding event and factor infusion Access to patient data by clinicians Alert function to warn patients and physicians of critical clinical events Duration: 3-12 months	Patient satisfaction	Feasibility Usage metrics	Very satisfied: 2 patients Moderately satisfied: 4 patients Not satisfied: 1 patient Nine patients continued the electronic documentation after the study. On-demand patients do not see any benefit in an electronic documentation system.
Vallee-Smejda et al, 2009 [19]	Advoy.com (internet-based electronic patient treatment record and communication system) Duration: 6 months	Adherence to recording Patient satisfaction	Usage metrics	Significantly more patients (29.4%) indicated satisfaction with electronic recording, as compared with paper records (6.7%). Electronic recording significantly improved patient adherence in recording mandatory treatment information. Electronic recording resulted in providing additional health data.
Leone et al, 2011 [20]	HeliTrax System handheld monitoring device Duration: 3 months	Perceived value of intervention	Usage metrics Ease of use Proficiency of generated reports	86% of patients rated higher value for electronic recording over traditional paper logging. Approximately 90% of patients rated the ease of tracking as good or excellent. Approximately 80% of patients rated the picture-taking capability and importance of that feature as good.
Broderick et al, 2012 [21]	Weekly SMS^c^ to monitor incidence of bleeding episodes Duration: 52 weeks	—	Response rate (proportion of weeks in which participants responded to the SMS) Cost	Response rate: 86.8% Small but significant decrease in response rate over 52 weeks Use of SMS is associated with high response rates and minimal expense and intrusion.
Mulders et al, 2012 [25]	E-learning (online course) Interactive multimedia program Education on home treatment of hemophilia Duration: 1 month	Knowledge on home treatment Practical skills: self-injection Self-efficacy		Significantly higher levels of theoretical knowledge and practical skills in the intervention group, as compared to the control group. No group difference in self-efficacy
Young et al, 2012 [22]	Electronic diary Internet-based entries submitted in real time Documented bleeding event, productivity, HRQoL^d^, and pain assessment Weekly contact by patient support liaison personnel	—	Usage metrics Degree of accuracy from validation	Adults: 1364 paper and electronic diary days Caregivers: 1165 paper and electronic diary days Exact agreement observed between electronic and paper records for 93% of the HRQoL scores reviewed

Breakey et al, 2013 [27]	“Teens Taking Charge: Managing Hemophilia Online” (interactive website to help patients transit from pediatric to adult care) Include hemophilia-specific education, self-managementstrategies, images, interactive animations, quizzes, and a glossary	Participants’ satisfaction Qualitative usability testing approach with audio-taped observation and semistructured interviews	User performance	Adolescent patients responded positively to the content and appearance of the website. Adolescent patients felt that it was easy to navigate and understand The multimedia components (videos, animations, and quizzes) enriched their experience.
Sholapur et al, 2013 [23]	EZ-Log Web Client (electronic diary)	Identify strengths and challenges of traditional vs electronic diaries	Usage metrics	Advantages: ease of use, improved accuracy, and less time consuming Disadvantage: Technical errors and inability to make corrections that require contact with the clinic Suggestions: Saving infusion history, incorporating barcode scanners
Breakey et al, 2014 [26]	Eight-module online program Interactive content Animations, illustrations, and knowledge quizzes Duration: 8-10 weeks	Disease-specific knowledge HRQoL Self-efficacy Self-management ability and transition readiness Program satisfaction	Not applicable	Significant improvement in disease-specific knowledge, self-efficacy, and transition preparedness in the intervention group Program informative, comprehensive, and easy to use
Hay et al, 2017 [13]	Haemtrack (electronic home treatment diary) interfaces with the UK Haemophilia Centre Information System and theNational Haemophilia Database Documented bleeding event, infusion log, pain assessment, and outcome	Adherence to electronic recording	Usage metrics	Electronic diary used by 90% of participating hemophilia treatment centers 72% (1923/2683) of patients used electronic diary, entering >17,000 treatments per month Adherence to reporting varied, and 55% of patients reported ≥75% of expected factor usage. No relationship between the patient’s age and the type of reporting medium preferred
Cuesta-Barriuso et al, 2018 [24]	Medtep Hemophilia online platform (electronic diary and reminder) Documented infusion log, physical activities, and bleeding event Unrestricted real-time access by clinicians to patient’s data Duration: 12 months	Adherence to prophylactic treatment HRQoL Perception of illness Joint health	Usage metrics	Two-thirds of patients consistently had above 80% adherence. Significant increase in treatment adherence from baseline to 1 month, 6 months, and 12 months after intervention Significant improvement in HRQoL and illness perception from baseline to 12 months. No change in joint health

^a^Not applicable.

^b^The primary objective of this study was to evaluate the efficacy of ReFacto, with the use of an electronic diary as the mode of documentation for bleeding events. Evaluating the effectiveness of electronic diary was an exploratory objective of this study.

^c^SMS: short message service.

^d^HRQoL: health-related quality of life.

## Discussion

### Principal Findings

This systematic review summarizes the existing literature that evaluated the efficacy of distance-delivered technologies for improving adherence to prophylactic infusion and electronic recording of bleeding events and for ameliorating functional outcomes among patients with hemophilia. Specifically, there were too few high-quality studies from which to draw strong conclusions in support of the use of telehealth interventions in this population. Despite the need for more RCTs with larger samples to validate our findings, preliminary evidence supports the feasibility and effectiveness of interventions delivered by telehealth for improving self-management and health literacy among this population, especially among patients with severe hemophilia who require regular prophylactic infusions. However, the collective evidence seems to suggest that the technical errors and complex technological operations are major patient-related barriers. Additionally, the significant effects of telehealth interventions on symptom management were not consistently established.

Over the past decades, advances in treatment modalities have reduced the mortality and morbidity associated with hemophilia. However, the life-long maintenance of a demanding treatment regimen requires excellent self-management skills from both patients and their caregivers. Telehealth has become increasingly popular for providing integrated care to patients with chronic diseases. For patients with hemophilia, telemedicine-delivered integrated care includes the remote provision of education to improve self-efficacy. As good record keeping is an important aspect of home-based hemophilia care, our review also identified evidence to support the use of telemonitoring in order to enable increased adherence to the recording and transfer of clinical information, such as bleeding episodes and infusion logs. It is also worth noting that a telehealth intervention should not be administered alone. A handful of included studies adopted multimodal components to complement the telehealth technology, such as preintervention training and regular personal contact with the patient during the study period [12,14,16,22]. This multimodal approach may potentially enhance the user’s proficiency, leading to increased acceptance and long-term adherence to the technology.

**Figure 2:**
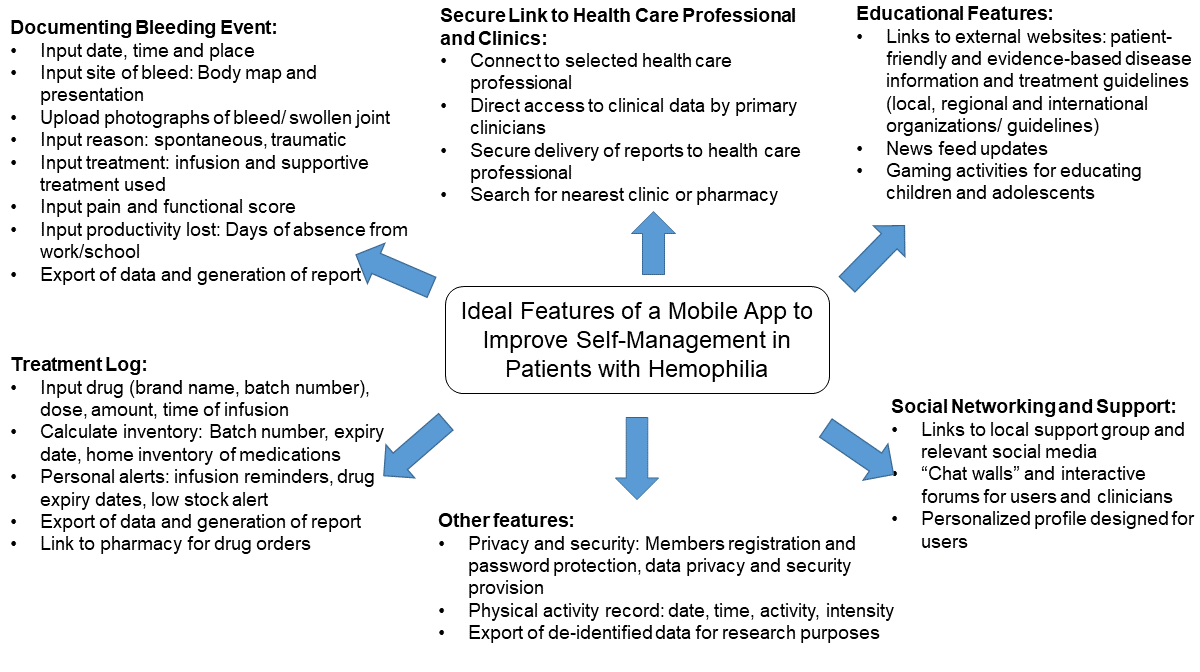
Ideal features of a mobile app to improve self-management in patients with hemophilia.

The growing popularity of mobile health (mHealth) over the past decade highlights the increasing trend involving the connection of patients with the world of digital health information via smartphones and other mobile devices [28]. One included study reported that that phone apps are associated with the most rapid reporting of bleeding episodes to the treatment center, with almost 40% of data being reported on the day of treatment and 70% reported within a week [13]. Based on the narrative syntheses in this review, we propose the features of an ideal mobile app for patients with hemophilia (Figure 2). This list comprises basic functions that allow the documentation of bleeding events and photographs, infusion logs, alerts, and a home inventory of medications. The comprehensive care of a patient with hemophilia also includes addressal of the patient’s psychological needs, and in this regard, interactive platforms (eg, chat walls and forums) that foster a supportive social network within the hemophilia community would be an attractive feature, although caution has to be taken to ensure good netiquette among users. To promote sustained usage and adherence, one must also consider applying the principles of behavioral science when designing a mobile app from a technical perspective [29,30]. Applications that support personalization, allow user-friendly data recording, and provide well-paced reminders may more effectively promote behavioral changes.

Such an app should also adopt a multimedia approach to update disease- and treatment-related information. In this context, gamification deserves more attention and study, particularly as a means to engage adolescents who are becoming increasingly dependent in health management [31]. Reliable authentication systems must also be implemented to protect the patients’ electronic health information, such as guidelines stipulated in the Health Insurance Portability and Accountability Act (HIPAA) in the United States or the Data Protection Act in the United Kingdom. Additionally, the ability to export de-identified data at the backend will provide a rich source of clinical data for research purposes.

### Clinical Implications and Direction for Future Research

Telehealth interventions can be used to provide quality health care to patients without readily accessible clinical services, such as those who reside in the inner city or rural areas. One study identified time, transportation difficulties, and the distance to the hematology clinic as the top barriers to obtaining care in the United States, especially for caregivers of children with hemophilia [32]. Beyond the reductions in travel costs, school-based telehealth programs may reduce the requirement for caregivers to secure time off from work and reduce the frequencies of emergency department and clinical visits. In developing countries, the lack of access to specialized clinics remains a major issue faced by patients who have recently initiated inhibitor therapy and require highly specific and closely monitored dosing. The introduction and systematic evaluation of telehealth programs in these areas could potentially expand patient access while reducing burdens such as travel required to receive specialty care, and improve monitoring, timeliness, and communication within the care continuum.

People with low levels of health literacy use more health care services, including visits to general practitioners, hospitals, and emergency care facilities [33,34]. This phenomenon may indicate that these patients bypass preventive care, adhere poorly to prophylaxis treatments, or are unable to make effective use of health care services [33]. A low level of health literacy is also associated with a reduced ability to take medications appropriately and a reduced adherence to chronic medication therapy. To address these issues, telehealth interventions can be implemented to promote motivation and ability among individuals by enabling access to understanding and processing of health-related information through improvements in cognitive and social skills. Built-in alerts and reminders within mobile apps may enhance adherence to prophylactic infusions. Of note, the three included studies involving online educational programs reported excellent outcomes in terms of improving patients’ self-efficacy and theoretical knowledge regarding disease management [25-27]. Such interventions were designed to be more engaging than traditional patient information leaflets or booklets by using more audiovisual information and interactions intended to demolish the literacy barrier. As hemophilia is a chronic disease characterized by complex care needs, an appealing telehealth-delivered educational intervention may play an important role in empowering adolescents as they transition to adulthood and take a more independent role in managing their health.

Cultural factors must be considered when selecting the most appropriate delivery of technology. Research has suggested that the levels of patient engagement and health literacy differ by race and ethnicity. Distance-delivered interventions for patients with hemophilia should include culturally tailored patient education programs and materials. For example, one qualitative study discussed the subjective illness experiences of patients with hemophilia in the United States and United Kingdom, with a particular focus on cultural and social contexts [35]. The authors found that patients in the United States tended to more strongly emphasize on existing support systems, such as relationships with health care practitioners or the cost of health insurance, whereas patients in the United Kingdom considered functional problems related to pain and disability more relevant in their everyday lives [35]. In addition to considering the involvement of cultural context in every aspect of the care continuum, the development of a telehealth intervention should also focus on the patients’ personal experiences of sickness and its effects on other relational contexts, such as family, school, or work. Most commercially available social networking apps currently used for patients with hemophilia were developed using English as the primary language. Therefore, future research should evaluate the needs of patients in non-English–speaking countries and thus elucidate the role of sociocultural variables in modifying the experience of this disease through advanced technology.

Telehealth interventions can potentially facilitate regional and international “teletwinning” to allow collaborative research and harmonization of data collection from patients with hemophilia [4]. One included study used a handheld computer diary platform to gather data on bleeding episodes from patients with severe or moderate hemophilia A in four Nordic countries (Denmark, Finland, Norway, and Sweden) [17]. The authors concluded that this platform not only improved adherence to reporting but also enabled the long-term postmarketing surveillance of treatment products by pooling data from different regions. In recent years, mHealth has emerged to augment specialized health care services delivered to underserved populations, especially in countries with high levels of health disparity such as India [36] and mainland China [37]. Teletwinning between developed and developing countries may bridge health care disparities and extend the highest standards of hemophilia care to all patients.

In addition to evaluating the efficacy, it is important to consider the cost-effectiveness of telehealth interventions for hemophilia management. Only one included study reported the costs associated with administering an SMS intervention from the economic perspectives of the participant and institution [21]. Compared to the traditional modes of health care delivery, new technologies will inevitably demand resources to develop, customize, initiate, and maintain the platforms and ensure the efficient achievement of the intended purposes. Data on the value and cost-effectiveness of such technologies would be indispensable if the eventual goal is to implement telehealth interventions in public health care systems on a wide scale. However, we acknowledge that such comparative studies may be methodologically difficult to conduct, as the hemophilia population is small and would require quantitative outcomes such as mortality and emergency department visits due to bleeding episodes, collected over a longer time horizon.

### Limitations

Hemophilia is a rare disease, and this review was limited by the inclusion of studies with a small sample size and lacking a control group. The wide variability in outcome measures and intervention regimens as well as the reliance on self-reported efficacy and satisfaction measures limited our ability to compare and draw conclusive results regarding the effects of the interventions. Heterogeneity in the assessment of health outcomes across studies also made it difficult to determine the effectiveness of the health technologies. Lastly, we acknowledge that even though a protocol was developed prior to the initiation of this review, it was not prospectively registered with any registries that facilitate public scrutiny. However, much effort has been dedicated to ensure strict adherence to the PRISMA (Preferred Reporting Items for Systematic Reviews and Meta-Analyses) guidelines and transparency and comprehensiveness in reporting. Given these limitations, our collective findings should be interpreted cautiously.

### Conclusions

To our knowledge, this is the first systematic review to evaluate the collective evidence of telehealth-delivered interventions for patients with hemophilia. Most interventions involved reminding patients to administer prophylactic infusions and documenting bleeding events and treatment logs. Although little explicit evidence is available, telehealth-delivered interventions could feasibly improve patients’ adherence to medication use and promote independence in disease management. Multimedia educational platforms appear to effectively enhance knowledge transfer and information utilization, particularly among adolescent and young adult patients. However, the sustainability of use and long-term adherence to the intervention remain uncertain. Given the complexity and resources involved in developing a mature and established system, support from a dedicated network of hemophilia specialists and data managers will be required to maintain the technology, improve adherence to prophylactic treatment and recording, and validate the electronic data locally. Future research should also involve RCTs with longer time horizons to investigate the effects of interventions delivered by telehealth on improved health outcomes and behaviors (eg, physical activity) among patients with hemophilia.

## Abbreviations

EPHPP: Effective Public Health Practice Project Quality Assessment Tool for Quantitative Studies
Hem: hemophilia
HemA: hemophilia A
HemB: hemophilia B
HIPAA: Health Insurance Portability and Accountability Act
HRQoL: health-related quality of life
IQR: interquartile range
ITT: immune tolerance treatment
NR: not reported
OS: observational study
RCT: randomized controlled trial
SMS: short message service
